# Normocytic Anemia as a Side Effect of Clozapine: A Case Report

**DOI:** 10.7759/cureus.67680

**Published:** 2024-08-24

**Authors:** Tambi I Isaac

**Affiliations:** 1 Department of Surgery, Wyckoff Heights Medical Center, Brooklyn, USA; 2 Medical Academy, Kabardino-Balkarian State University, Nalchik, RUS

**Keywords:** antipsychotic side effect, hematological abnormalities, clozapine side effect, schizophrenia, anemia due to clozapine, clozapine-induced anemia

## Abstract

This brief case report aims to shed light on an uncommon blood-related side effect potentially associated with clozapine use, an atypical antipsychotic primarily prescribed for treatment-resistant schizophrenia.

A 70-year-old white male with a significant past medical history of schizophrenia controlled with clozapine was evaluated over approximately two months. Regular blood work was conducted to monitor his absolute neutrophil count, which is known to drop while on clozapine. During his stay, it was noticed that his hemoglobin levels were declining without a clear reason. Through the method of exclusion, the most common causes of anemia were ruled out, and it was determined that the patient's anemia was secondary to clozapine, a side effect that is not commonly reported.

The precise mechanism by which clozapine affects hemoglobin levels remains unclear. However, some studies suggest potential direct bone marrow toxicity. This is supported by the rapid improvement in hemoglobin observed after clozapine discontinuation in this case.

This case highlights a potential association between clozapine use and normocytic anemia. It emphasizes the significance of regular blood work monitoring not only for the absolute neutrophil count but also for hemoglobin. This rare case underscores the importance of considering red blood cell parameters in patients on clozapine, offering insights that go beyond the commonly reported agranulocytosis side effect and potentially expanding awareness of adverse hematological effects associated with this antipsychotic medication. Further research is crucial to unravel the underlying mechanisms and establish definitive causality.

## Introduction

Anemia is a condition that is characterized by a deficiency in the number of red blood cells (RBCs) or the amount of hemoglobin they contain which is essential for carrying oxygen to the tissues; several factors and causes are known to cause anemia including iron deficiency, vitamin deficiencies (folate, B12), chronic diseases, blood loss, bone marrow and stem cell problems, hemolysis, medication side effects, and genetic conditions. In addition, anemia is classified based on the size of RBCs, which is indicated by mean corpuscular volume (MCV), into microcytic anemia (small RBC size), normocytic anemia (normal RBC size), and macrocytic anemia (large RBC size). Normocytic anemia is when the average size of RBC is within the normal range (which is 80-100 femtoliters); however, the overall number of RBC and the amount of hemoglobin are lower than normal. Clozapine, an atypical antipsychotic, primarily prescribed for treatment-resistant schizophrenia [1], is notably linked to agranulocytosis, a decrease in the number of granulocytes, a type of white blood cells that fight infections [2,3], among other side effects. This brief case report aims to shed light on an uncommon blood-related side effect, namely, normocytic anemia, that is potentially associated with clozapine use [4,5].

## Case presentation

The patient is a 70-year-old white male, with a significant past medical history of hypertension, osteoarthritis, benign prostatic hyperplasia, anxiety, schizophrenia, and left total hip arthroplasty. He was in assisted living and was transferred to the nursing home for rehabilitation following a left total hip arthroplasty in a local hospital.

His medication regimen comprised of the following: amlodipine 5 mg, one tablet daily; clozapine 100 mg, three tablets at bedtime; vitamin D 50,000 units, once a week; tamsulosin 0.4 mg, once at bedtime; paroxetine 20 mg, one tablet daily; and ferrous sulfate 325 (65 Fe) mg, one tablet daily.

The patient was admitted to the nursing home for rehabilitation two weeks after the surgery; his initial blood work unveiled abnormalities, notably anemia as shown in Table 1.

**Table 1:** Initial CBC RBC: red blood cell; Hg: hemoglobin; Ht: hematocrit; MCV: mean corpuscular volume; RDW: red cell distribution width; CBC: complete blood count

Test	Range	Reference range
RBC	2.98	4.50-5.90 × 10^6^/uL
Hg	8.6	13.3-17.7 g/dL
Ht	26.0	40.0-50.0%
MCV	87.2	80.0-100.0 fL
RDW	13.3	11.5-14.5%

As shown in Table 2, the patient's baseline hemoglobin following arthroplasty at the hospital was 13.9.

**Table 2:** Baseline CBC after arthroplasty RBC: red blood cell; Hg: hemoglobin; Ht: hematocrit; MCV: mean corpuscular volume; RDW: red cell distribution width; CBC: complete blood count

Test	Range	Reference range
RBC	4.79	4.50-5.90 × 10^6^/uL
Hg	13.9	13.3-17.7 g/dL
Ht	40.1	40.0-50.0%
MCV	83.7	80.0-100.0 fL
RDW	13.4	11.5-14.5%

There was no report of blood loss during the surgery or blood in the urine or stool. The patient did not experience symptoms related to infection such as fever, chills, cough, sore throat, and dysuria. He did not have any skin ulcerations or decubitus pressure ulcers which are prevalent in nursing home patients. Despite low hemoglobin, the patient was asymptomatic. The physical exam was only significant for bilateral pallor conjunctivae. The patient is independent in daily activities and able to ambulate with a roller walker.

Diagnostic work-up

The work-up plan was focused on excluding other possible causes of anemia. The patient's urinalysis and fecal occult blood test resulted negative. There was no clear site of bleeding. The patient's iron levels, red cell distribution width (RDW), total iron-binding capacity (TIBC), and ferritin levels are shown in Table 3.

**Table 3:** Iron deficiency anemia work-up RDW: red cell distribution width; TIBC: total iron-binding capacity

Test	Range	Reference range
Iron	65	65-175 ug/dL
Ferritin	63	7.3-270.7 ng/mL
RDW	13.2	11.6-14.4%
TIBC	260	250-425 ug/dL

While the patient was in the nursing home, his hemoglobin declined from 13.9 g/dL to 8.6 g/dL. The patient was on clozapine 100 mg three times daily for an unknown period. He had a good appetite and no history of dysphagia or malabsorption, in addition to normal iron and ferritin levels (as shown in Table 3), making iron deficiency anemia secondary to nutritional deficiency minimal on the differential scale.

Treatment and outcome

**Figure 1:**
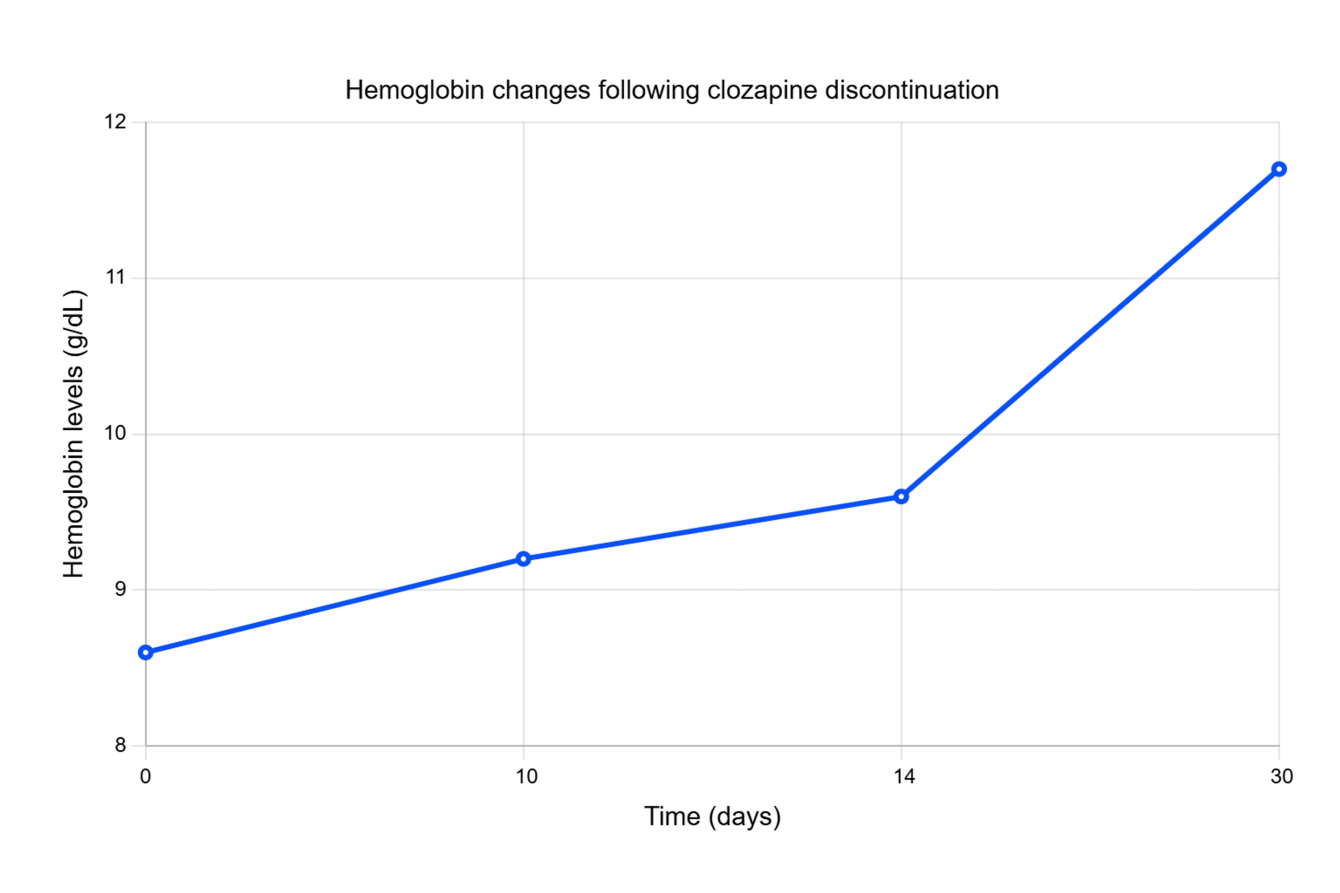
Hemoglobin changes since the intervention (discontinuation of clozapine)

Due to the lack of comprehensive information regarding the patient's schizophrenia management with clozapine, a cautious approach was taken, initiating the discontinuation of clozapine. The patient's symptoms were vigilantly monitored with regular blood work evaluations. Three weeks post-clozapine cessation, significant improvements in hemoglobin levels were noted as illustrated by the line graph below (Figure 1).

However, psychosis-like symptoms resurfaced, prompting the introduction of risperidone 2 mg daily. Subsequently, there was a notable improvement in anemia and psychotic symptoms, leading to the patient's discharge after completing the rehabilitation program.

The patient's discharge medications were as follows: aspirin 81 mg, one tablet daily; ferrous sulfate 325 (65 Fe) mg, one tablet daily; lorazepam 1 mg, every eight hours; losartan potassium 100 mg, one tablet daily; metoprolol succinate ER 25 mg, one tablet daily; vitamin D 5000 unit, one capsule weekly; and risperidone 2 mg daily.

## Discussion

The exact mechanism by which clozapine affects hemoglobin levels remains unclear, as demonstrated in two different studies [4,5]. The pathogenesis may involve direct bone marrow toxicity caused by clozapine or its metabolites, which is suggested by the rapid improvement in hemoglobin levels following clozapine discontinuation (approximately 30 days in this case). Alternatively, it may be related to a drug-antibody complex, as indicated in another study [4]. Normocytic anemia as a potential side effect of clozapine is rare, as evidenced by the limited number of case reports. It is possible that certain individuals are more predisposed to developing anemia while on clozapine compared to the general population, but this hypothesis requires further investigation with a larger number of similar cases. Despite the exclusion of other common causes of anemia through the diagnostic work-up (i.e., iron deficiency, vitamin B12/folate deficiency, anemia of chronic disease, and chronic blood loss), this study lacks a crucial diagnostic method (bone marrow biopsy) that could have provided additional insights into the pathogenesis. This limitation is due to the patient's environment (nursing home) and the disruption of continuity of care (patient discharge).

## Conclusions

This case highlights a potential association between clozapine use and normocytic anemia. It emphasizes the significance of regular blood work monitoring not only for the absolute neutrophil count but also for hemoglobin, which adds a novel dimension to our understanding of clozapine-induced hematological abnormalities. This rare case underscores the importance of considering RBC parameters in patients on clozapine, offering insights that go beyond the commonly reported agranulocytosis side effect and potentially expanding awareness of adverse hematological effects associated with this antipsychotic medication. Further research is crucial to unravel the underlying mechanisms and establish definitive causality.
